# The *DCDC2* deletion is not a risk factor for dyslexia

**DOI:** 10.1038/tp.2017.151

**Published:** 2017-07-25

**Authors:** T S Scerri, E Macpherson, A Martinelli, W C Wa, A P Monaco, J Stein, M Zheng, C Suk-Han Ho, C McBride, M Snowling, C Hulme, M E Hayiou-Thomas, M M Y Waye, J B Talcott, S Paracchini

**Affiliations:** 1 Walter and Eliza Hall Institute of Medical Research & Melbourne University, Parkville, VIC, Australia; 2 School of Medicine, University of St Andrews, St Andrews, UK; 3 School of Biomedical Sciences, The Chinese University of Hong Kong, Shatin, Hong Kong; 4 Wellcome Trust centre for Human Genetics, University of Oxford, Oxford, UK; 5 Department of Physiology, University of Oxford, Oxford, UK; 6 Department of Psychology, The University of Hong Kong, Hong Kong, Hong Kong; 7 Department of Psychology, Shatin, NT, The Chinese University of Hong Kong, Shatin, Hong Kong; 8 Department of Experimental Psychology and St John's College, University of Oxford, Oxford, UK; 9 Department of Education, University of Oxford, Oxford, UK; 10 Department of Psychology, University of York, York, UK; 11 The Nethersole School of Nursing, The Chinese University of Hong Kong, Shatin, Hong Kong; 12 Aston Brain Centre, School of Life and Health Sciences, Aston University, Birmingham, UK

## Abstract

Dyslexia is a specific impairment in learning to read and has strong heritability. An intronic deletion within the *DCDC2* gene, with ~8% frequency in European populations, is increasingly used as a marker for dyslexia in neuroimaging and behavioral studies. At a mechanistic level, this deletion has been proposed to influence sensory processing capacity, and in particular sensitivity to visual coherent motion. Our re-assessment of the literature, however, did not reveal strong support for a role of this specific deletion in dyslexia. We also analyzed data from five distinct cohorts, enriched for individuals with dyslexia, and did not identify any signal indicative of associations for the *DCDC2* deletion with reading-related measures, including in a combined sample analysis (*N*=526). We believe we conducted the first replication analysis for a proposed deletion effect on visual motion perception and found no association (*N*=445 siblings). We also report that the *DCDC2* deletion has a frequency of 37.6% in a cohort representative of the general population recruited in Hong Kong (*N*=220). This figure, together with a lack of association between the deletion and reading abilities in this cohort, indicates the low likelihood of a direct deletion effect on reading skills. Therefore, on the basis of multiple strands of evidence, we conclude that the *DCDC2* deletion is not a strong risk factor for dyslexia. Our analyses and literature re-evaluation are important for interpreting current developments within multidisciplinary studies of dyslexia and, more generally, contribute to current discussions about the importance of reproducibility in science.

## Introduction

Dyslexia, or reading disability (RD), is a common neurodevelopmental disorder, with prevalence estimates ranging between 5–10% among school-age children in most countries.^1, 2^ While a significant genetic component has been firmly established for this condition, only a small number of specific genetic factors have been identified to date.^3^ These include variants within the *DCDC2* gene, located on chromosome 6 (at 6p22.3). Single-nucleotide polymorphisms (SNPs) within *DCDC2* showed association with both dyslexia and reading abilities in independent studies,^4, 5, 6, 7, 8, 9, 10^ but the functional mechanisms underlying these associations remain unclear. Attention has increasingly focussed on an intronic deletion of 2,445 bp within *DCDC2* as a potential functional element (Table 1).

The original study describing the *DCDC2* deletion reported associations between this locus and quantitative measures.^4^ However, such associations were detected only when the deletion was analyzed in combination with a pool of 10 rare alleles of a short tandem repeat (STR; BV677278) marker at the same locus.^4^ Analyses of the deletion combined with the STR alleles, failed to replicate the association with dyslexia as a binary category.^13, 14^ A separate study reported an association with a composite memory score, but the association appeared to be driven by one particular STR allele (allele 10 with frequency of 5%) rather than the deletion itself.^15^ This STR has been suggested to be a regulatory element.^11, 16^ Powers *et al.* (2013)^11^ reported that two 6-SNP haplotypes (allele frequency<5%)) were associated with reading and language phenotypes in a large epidemiological sample (the Avon Longitudinal Study of Parents and Children (ALSPAC)) stratified for a severe phenotype (⩾2 s.d. below the mean on a phoneme-deletion task). This stratification resulted in the comparison of 89 cases with the remaining 5,225 individuals who scored above the −2 s.d. diagnostic threshold. These two rare haplotypes were in linkage disequilibrium (LD) with alleles 5 and 6 of the STR. In a follow up study, allele 5 (3.6% frequency) and 6 (4.7% frequency), but not the deletion, showed association with reading and language measures.^12^ Meng *et al.* (2011)^16^ showed that STR alleles 3, 4 and 5 have differential effects on gene expression regulation. In summary, there is no clear consensus as to which STR alleles are associated with dyslexia and regulate gene expression. Rare alleles, such as the STR variants, can increase the likelihood of false positives because their analysis is typically conducted in very small samples.^17^ Up to 40 alleles have been described for the STR,^11, 13^ and therefore it is expected that some alleles would lead to significant *P*-values purely by chance.

The deletion has also been analyzed without the inclusion of the STR marker. Weak associations have been reported for various reading-related traits with significance values in the range of 0.02<*P*<0.05^14, 15, 18^ (Table 1; Supplementary Table 1). Lack of replication has also been reported.^12, 14^ Wilcke *et al.* (2009) reported the most compelling association to date (*P*<0.01) in a case/control study of a German cohort (Table 1). In this sample, the deletion had a frequency of 10% in cases (8% in severe cases) and 4% in controls. This lower frequency in the controls, however, is not consistent with what is observed in other studies of European samples. In fact, the deletion had a frequency of 8.6% among the parents of probands with dyslexia in a different German sample.^13^ A frequency of 9% was reported for both cases with dyslexia and controls in a UK cohort,^14^ similar to the 8.3% frequency reported in a UK epidemiological sample.^12^

Taken together, these studies do not support a role of the *DCDC2* deletion as a specific risk factor for dyslexia. Nevertheless, the *DCDC2* deletion is becoming increasingly used in research aimed at identifying the neuronal correlates of dyslexia. For example, the deletion has been suggested to directly affect the development of brain structures relevant to language and reading, through quantitative imaging studies of anatomical variability in healthy individuals.^19, 20^ The deletion has also been linked to functional mechanisms, such as reduced sensitivity to visual coherent motion.^21, 22^ Perception of motion is an important component of visual development, for which impairment has been demonstrated to occur in several developmental disorders,^23^ including dyslexia.^24^ Observed associations between visual motion sensitivity and reading skills contributed to the development of the magnocellular hypothesis for dyslexia.^25^ However, motion perception deficits may be restricted to subgroups of individuals with dyslexia, and typically those with the most severe phenotypes who might also present comorbidities with other developmental disorders.^26^ Cicchini *et al.*^22^ reported that a decreased ability to detect and discriminate visual motion correlated with the presence of the *DCDC2* deletion and suggested that motion sensitivity was more strongly associated with the presence of the deletion than with the presence of dyslexia. A major limitation of these imaging and behavioral studies, however, is the very small sample size, which suffers from both low power of detecting real effects as well as the increased chance of generating false positives.^17^

The aim of the present study was to provide additional data to evaluate the role of the *DCDC2* deletion as a contributor to risk for dyslexia and impairment on related measures. In addition to a re-evaluation of the published literature (Table 1), we report new association analyses for the deletion in four distinct cohorts characterized for reading measures, including, for the first time, to our knowledge, a cohort of Asian origin. We also present a replication analysis for association between the *DCDC2* deletion and a measure of visual motion sensitivity. The evidence we have gathered does not support an association between the *DCDC2* deletion and dyslexia or quantitative measures of reading abilities. This work is important for the interpretation of a developing body of literature and for framing the direction of future research studies of dyslexia. Moreover, our results are relevant to the current discussion about the importance of reproducibility in science,^27^ including the observation that it may be very easy to canonize weak findings as scientific facts unless a substantial number of negative findings are published.^28^

## Materials and methods

### Study participants

Our analysis included four distinct UK cohorts, referred to here as the Oxford Family Dyslexia cohort, Oxford Cases Dyslexia Cohort, Aston Dyslexia cohort, York cohort, and a fifth cohort, the Hong Kong cohort from the Chinese-English Twin Study of Biliteracy (Table 2).

The Oxford Family Dyslexia cohort was recruited by research clinics in Oxford and Reading. Genotype and phenotype data were available for 219 families (445 siblings, age range 6–27 years). Parental genotypes were also available. The same research clinics recruited the Oxford Cases Dyslexia Cohort, a collection of 272 unrelated individuals (age range 8–18 years). The psychometric measures used to assess these cohorts included the British Ability Scales (BAS) single-word reading (READ) and spelling (SPELL) tests,^29^ irregular word reading (OC-irreg)^30^ and phonological decoding (PD).^30^ An orthography measure using the forced choice task (OC-choice)^31^ and phoneme awareness (PA)^32^ scores were available for the Family cohort. Both cohorts have been described before^33^ and the Family cohort was also previously analyzed for association between the *DCDC2* deletion and reading abilities.^14^ Here, we analyzed the Oxford Family cohort for association with a visual motion (VMOT) measure described previously^34^ (See also Supplementary Materials for details) and as part of a combined sample (see below).

The Aston Dyslexia cohort includes 105 unrelated individuals, recruited through the Aston Dyslexia and Developmental Assessment Unit in Birmingham, UK (age range 7–16 years). Children attending this educational clinic have been referred for assessments of reading difficulties by local schools. On the basis of their educational assessments, children are invited back to participate in research studies. The phenotypes obtained for this cohort included single-word reading (READ) and single-word spelling (SPELL), measured by standardised assessments including using the Wechsler Individual Achievement Test,^35^ the British Abilities Scales, and the Test of Word Reading Efficiency (TOWRE).^36^ Sub-scales of TOWRE were used to measure pseudo-word reading (or phonological decoding, TOWRE_PD) and sight word efficiency (TOWRE-SWE).

The York cohort is a longitudinal cohort designed to study language and literacy development^37^ and has been analyzed previously for genetic studies.^38^ DNA was available for 103 families and a total of 318 individuals. Of these, phenotype data were available for *N*=103 probands and *N*=41 siblings. Phenotypes were available from the same children at different time points. Reading abilities were measured at the ages of 4½, 5½, 6 and 8 years, representing the years when reading skills are in ascendancy. At age 5½ years, the measure was word reading from the York Assessment of Reading Comprehension (READ);^39^ at age 6 years, the measures were word reading efficiency from the Test of Word Reading Efficiency Word (TOWRE_READ) and Pseudoword (TOWRE_PD) tests^36^ and spelling from the Wechsler Individual Achievement Test (WIAT-SPELL);^35^ at age 8 years the measures were irregular word reading (IWR) from the Diagnostic Test of Word Reading Processes (DTWRP),^40^ phonological awareness measured by a phoneme-deletion task (PA) and rapid automatic naming (RAN).^41^ The factor scores were latent variables derived following confirmatory factor analysis: a phonology factor score (PHON_FS) was derived from word recall and phoneme awareness tasks at 4½ years and from phoneme awareness (deletion) at ages 5½, 6 and 8 years; a literacy factor score (LIT_FS) was derived from measures of single-word reading, reading accuracy, word and non-word reading fluency and spelling at age 8 years. At age 8 years, children were assigned to a formal diagnosis of dyslexia when they scored >1.5 s.d. below the control group mean on the literacy composite measure. This approach identified *N*=18 children with dyslexia, including *N*=9 who also had language impairment (LI). Another *N*=11 had LI only and *N*=72 children were classified as typical developing (TD).

Exclusion criteria for the UK cohorts were non-European ethnicity, signs of other neurological conditions in the probands or performance IQ<80.

The Hong Kong cohort included 220 unrelated individuals who were not selected for reading difficulties (age range 6–10.5 years old).^42^ Study participants were recruited either as singletons (*N*=104) or as twin pairs with only one twin per pair retained for the study (*N*=116). All participants speak Cantonese as their first language and have not been diagnosed with either intellectual disabilities or neurological conditions. Three reading-related phenotypes were available from a standardized test battery described previously: Chinese Word Reading (CWR), Chinese one-minute (COM) word reading and Chinese Digit Rapid Automatized Naming (CDRAN).^43^ Mean scores are shown in Supplementary Table 2.

DNA was extracted from blood or buccal swabs using standard procedures, and from saliva using Oragene kit (prepITL2P) (DNA Genotek, Ottawa, Canada). Ethical approval was obtained from the local ethical research committees, including the Oxfordshire Psychiatric Research Ethics Committee, the NHS Research Ethics (Yorkshire & The Humber – Humber Bridge), the University of York Department of Psychology Ethics Committee and the Aston University Ethics Committee, and the Joint Chinese University of Hong Kong-New Territories East Cluster Clinical Research Ethics Committee. Written informed consent was obtained from all participants and/or their caregivers.

### Genotype and statistical analysis

Genotype data were generated using a previously described protocol^4^ and checked for Mendelian errors and deviation from Hardy-Weinberg equilibrium using PEDSTATS.^44^ Family-based cohorts were tested using the Quantitative Transmission Disequilibrium Test (QTDT) under the total association model^45^ after estimating the identical-by-descent (IBD) sharing with MERLIN.^46^ No population stratification was observed for the UK cohorts following analysis of genome-wide genotype data.^47, 48^ Singletons analysis was conducted with PLINK (Purcell *et al.*, 2007) under an allelic model in each cohort as well as in combined samples including the Oxford Cases cohort, the Aston cohort and the probands of the Oxford Family cohort. Analysis was also conducted in a subgroup derived from this combined sample for meeting a stringent criteria of dyslexia defined by a score below −1.5 s.d. from the normative population mean for a single-word reading test. Power calculations were conducted with the genetic Power Calculator^49^ (Supplementary Material).

## Results

### The *DCDC2* deletion and reading abilities

The frequency of the deletion ranged 6.1–9.8% in the UK cohorts (Table 2), which is consistent with the range reported previously, including that for UK controls (9% Table 1). Differences in frequency across the cohorts and in specific subgroups likely resulted from sampling bias and illustrates how small samples can potentially affect this type of analysis. For example, the longitudinal design of the York cohort enabled the identification of children with dyslexia on the basis of very detailed assessments. The overall frequency of the deletion, across the 103 probands was 7.7%, similar to that observed in the parents (7.4%, *N*=174). On the basis of the final diagnosis, the deletion had a frequency of 10.7% (*N*=72) in the TD group compared to 5.5% in the RD (*N*=18) group (Table 2). The deletion frequency in a combined group of individuals (*N*=203) from the UK cohorts meeting a stringent definition of dyslexia (READ<−1.5 s.d. from the standardised population mean) was 9.6%. Strikingly, the deletion had a frequency of 37.6% (*N*=220; Table 2) in the Hong Kong cohort which was sampled from a population with typical literacy achievement (Supplementary Table 2). Given the substantial difference in allele frequency between the UK and Hong Kong population we queried the data from the 1000 Genomes project.^50^ The deletion (ID=Esv3608367) presents a global frequency of 11.5% across 2504 individuals, and consistently with our data, shows a frequency of 8.05% and 33.53% in the European and East Asian populations (Table 2; Supplementary Table 3). The frequency observed in the European population is important in interpreting the results by Wilcke *et al.* (2009) that were based on comparisons with a control cohort of German origin that presented a deletion frequency of 4% (Table 1).

The *DCDC2* deletion was tested for association with quantitative reading-related measures in the Oxford Cases, Aston, York and Hong Kong cohorts. The *DCDC2* deletion was tested for association with reading measures in the Oxford Family Dyslexia cohort previously;^14^ no associations were detected in the entire cohort, and only marginally significant associations were observed in the subgroup of 126 families selected for severity of the phenotype (Supplementary Methods and Supplementary Table 1). We then conducted quantitative association analyses in the combined sample including individuals with directly comparable phenotypes. We used a combined sample including probands from the Oxford Family cohorts and the Oxford Cases (*N*=425) which had four comparable phenotypes. We then analyzed this sample with the addition of the Aston cohort (*N*=526) which was characterised with the same READ and SPELL phenotypes. In both combined samples we analyzed subgroups of individuals who met a stringent definition of dyslexia defined by a score below -1.5 s.d. from the standardised mean for READ of the population. No association was detected with any of the phenotypes tested (Supplementary Table 4).

### The *DCDC2* deletion and visual motion

The distribution of VMOT thresholds (threshold=1/sensitivity) approximated normality across *N*=701 individuals (including individuals for whom genotype data were not available) with a mean of 18.1 (s.d.=12.2) in the Oxford Dyslexia cohort. VMOT thresholds showed a small, but statistically significant correlation with READ (*r*=−0.16; one-tailed *P*=1.0 × 10^−5^), indicating that lowered motion sensitivity is associated with poorer reading scores.

We did not identify patterns of association between the *DCDC2* deletion and VMOT thresholds, either in the whole cohort or in the same subset of 126 families selected for severity as described previously^14^ (Supplementary Table 1 and Supplementary Material). The subgroup analysis has the limitation of reducing the power of the analysis by decreasing the sample size, but was aimed at addressing the alternative hypothesis that the effect of the deletion on visual motion abilities is restricted to individuals within the lower tail of the normal distribution of reading ability.^26^

We stratified the READ and VMOT scores for the *DCDC2* deletion genotypes across all siblings and for the probands only (Table 3). As expected, the mean scores for the probands showed lower performance for both READ and VMOT compared to the whole data set, but their raw scores were similarly distributed across the three genotype groups (Supplementary Figure 1). Accordingly, genetic association analysis run under different linear models (dominant, recessive and genotypic conducted using PLINK) did not show any trends of association (data not shown).

## Discussion

With this study, we aimed to investigate the role of the *DCDC2* deletion in contributing to dyslexia or measures of reading and component skills. Our work was prompted by recent studies that based their hypotheses and research designs on the assumption that the *DCDC2* deletion is an established susceptibility factor for dyslexia. However, as our re-evaluation of the literature shows, the accumulated evidence in support of this assumption is statistically weak (Table 1). Most prior studies either did not show association with the deletion^4, 12, 13, 14^ or showed only moderate signals of association (0.01<*P*-values<0.5)^15, 18, 51^ (Table 1). This latter group included also our previous study that reported a weak association signal (*P*=0.04) with one phenotype (IWR) in a subgroup of families (Supplementary Table 1). Only one study reported a significantly higher frequency of the deletion in cases with dyslexia (10%) compared to controls (4%).^7^ However, through comparisons with other studies^12, 13, 14^ and with the data derived from the 1000 Genome project for European reference populations (Supplementary Table 3), we suggest that the low frequency reported in the control cohort analyzed by Wilcke *et al.*^7^ most likely resulted from an artifact. Stronger association signals were observed only when the deletion was combined with different rare STR alleles.^4, 12, 15^ We did not test the STR in this study because our focus was to address the role of the *DCDC2* deletion specifically as a reliable marker for dyslexia. Our goal was to verify the theoretical foundation of the design of imaging^19, 20^ and behavioral^21, 22^ studies for which the deletion alone, and not in combination with STR alleles, has been used as a dyslexia marker. These studies suggested that the deletion is directly associated with structural brain changes and sensory deficits regardless of the presence of dyslexia, implying a causative effect for this genetic marker. Furthermore, the STR harbors at least 40 (mostly extremely rare) alleles, making it challenging to distinguish genuine signals from noise and to test specific hypotheses. In fact, associations so far have been reported for different STR alleles (i.e. deletion+pool of rare alleles, allele 10, allele 5 and allele 6; Table 1).

We tested the deletion for association with reading abilities in four cohorts from the UK analysed both separately and as combined samples, including analysis in a subgroup meeting stringent criteria for dyslexia. The Oxford Family Dyslexia cohort, analyzed previously,^14^ was re-analyzed here as part of this approach and for association with a visual motion test. Furthermore, we tested a cohort recruited in Hong Kong, which represents the first cohort of non-European ancestry to be analyzed for this marker in the context of reading abilities. None of our analyses showed any statistically (or nominally) significant associations between the deletion and reading measures. Power calculations indicate that our largest sample used for genetic analysis (*N*=526) has the power to detect only relatively large effects (>1.5% of the phenotypic variance; Supplementary Material). Although this is larger than what would generally be predicted for common genetic factors contributing to complex traits, our data are important for the interpretation of the current literature. The analysis in the Hong Kong cohort revealed a frequency of 37.6% which is consistent with frequency data reported for the 1000 Genomes Project Asian populations. The Hong Kong cohort is representative of the general Hong Kong population of Chinese origin and is not enriched for individuals presenting reading difficulties, as demonstrated by the distribution of their language and reading abilities scores (Supplementary Table 2). It is debated whether dyslexia in Chinese is the manifestation of a different deficit compared to European populations as a result of culture.^52^ However both behavioral^53^ and neuroimaging^54^ studies predict commonalities in the neuronal markers for dyslexia that can be dissociated from language-specific effects in both English and Chinese individuals. The incidence of dyslexia in Hong Kong, similar to the UK populations, is about 10%.^2^ However, visual processing may be more relevant for decoding Chinese compared to alphabetic scripts because of the complex visual composition of Chinese characters.^55^ The role of the visual magnocellular pathway in reading has been suggested to be particularly relevant for decoding Chinese scripts.^56^ The visual motion perception tests that compared groups on the basis of the *DCDC2* deletion genotypes were designed to assess the role of this pathway in dyslexia.^21, 22^ Genetic association analysis for VMOT measures in the Hong Kong cohort, or other Chinese speaking groups, would allow direct comparisons with these studies.

We tested for effects of the deletion on a visual motion task (Table 3; Supplementary Table 1) in the Oxford Dyslexia cohort and found no associations, thereby failing to replicate the findings by Cicchini *et al* (2015),^22^ albeit we used a different version of the VMOT test (see Supplementary Material for details). Cicchini and colleagues used a contrast sensitivity paradigm with a very short presentation time that varied the contrast of a moving stimulus to the detection threshold. Our VMOT task involved much longer presentation times for random dot kinematograms (RDK) in which the signal to noise ratio was manipulated to the detection threshold. Although we cannot directly compare the two tasks, the observation that the probands in our sample have lower sensitivity to motion compared to the entire sample (Table 3) provides cross-validation of previous work with this task in dyslexia^57^ and as a correlate with reading skills in the normal population.^34^ The sample we used for the VMOT analysis was substantially larger (*N*=374 non-carriers, *N*=66 heterozygotes; *N*=6 deletion homozygotes; Table 3) compared to the analysis reported by Cicchini and colleagues in 11 deletion carriers and 10 non-carriers. Therefore, while we cannot exclude an effect dependent on specific phenotypes or of smaller size, our study provides a new data set for the interpretation of previously reported results. Regardless of the differences in the tests used, our genetic analysis does not support the design of behavioral studies based on the stratification for *DCDC2* deletion genotypes.

While the *DCDC2* deletion is interesting for the potential regulatory effect, other *DCDC2* markers, mainly SNPs, showed more consistent associations across independent studies.^4, 5, 6, 7, 8, 9, 10^ Our current study, therefore, does not undermine the overall evidence in support of *DCDC2* as a candidate gene for dyslexia or reading abilities.

Replication studies are essential but present particular challenges. The acquisition of cognitive measures is only possible through expensive and time consuming one-on-one sessions, making it extremely difficult to obtain large sample sizes. Heterogeneity of phenotypic and behavioral measures makes direct comparison across studies very difficult. Overall, these observations reinforce the importance of collecting high quality cognitive data in general population samples to enable follow up studies of genetic associations reported for neurodevelopmental disorders.^58^

In summary, through both a re-evaluation of the published literature and new genetic association analyses, our results show that there is no strong evidence in support of the *DCDC2* deletion as a risk factor for dyslexia. These data are important for guiding the future direction of dyslexia research and more generally highlight the caveats associated with over-generalizing from analyses conducted in small sample sizes and from misinterpretations of the literature. Most importantly, we provide a useful example of the importance of publishing null results toward avoiding the potential of canonizing weak evidence as fact.

## Figures and Tables

**Table 1:** Previous studies that analyzed the *DCDC2* deletion for association with dyslexia

*Reference*	*Country*	*Sample*	*Type of analysis*	*Allele*	*Quantitative phenotype*	P*-value*	*Deletion Frequency*
Meng *et al.*, 2005	USA	153 nuclear families	Quantitative	Deletion+STR rare alleles	Homonym Choice	0.00002	8.5%
					Untimed PIAT Word Recognition	0.023	
					Word Recognition	0.04	
					OC	0.0035	
					OC	0.0385	
							
Harold *et al.*, 2006	England	264 nuclear families	Quantitative	Deletion		Not associated	10%
		126 severe families subgroup			IWR	0.03	9%
					READ	0.04	
					PA	0.05	
	Wales	350 cases	Categorical	Deletion		Not associated	9% cases
		273 controls		Deletion+STR rare alleles		Not associated	9% controls
							
Brkanac *et al.*, 2007	USA	191 trios	Quantitative	Deletion	SWE	0.05	7.5%
					Spelling	0.04	
							
Ludwig *et al.*, 2008	Germany	396 trios	Categorical	Deletion+STR rare alleles		Not associated	8.6%
							
Wilcke *et al.*, 2009	Germany	72 cases	Categorical	Deletion		*P*<0.01	10% cases
		25 severe cases					8% severe cases
		184 controls					4% controls
							
Marino *et al.*, 2012	Italy	303 nuclear families	Quantitative	Deletion	SWR	0.05	6%
				Deletion	PD	0.04	
				STR allele 10	Memory Composite Score	0.00001	
				Deletion+STR rare alleles	Memory Composite Score	0.00001	
							
Powers *et al.*, 2016	UK	89 cases	Categorical	Deletion	Severe RD	Not associated	8.3%
		4428 controls^a^			Severe LI		

Abbreviations: IWR, irregular word reading; LI, language impairment; OC, orthographic coding; PA, phonological awareness; PD, non-word reading; PIAT, Peabody individual achievement test; READ, single-word reading; RD, reading disability; SWE, sight word efficiency.

a The samples sizes were estimated by combining information from different studies;^11, 12^ these studies reported association between the 5 and 6 STR alleles with reading-related measures.

**Table 2:** Frequency of the *DCDC2* deletion reported in this study

*Cohort*	*Structure*	*Recruitment*	*DCDC2 deletion frequency*
Oxford Family Dyslexia	219 families	Clinical—children with reading difficulties	8.9% parents (*N*=414) 9.8% probands (*N*=153)^a^
			
Oxford Cases Dyslexia	272 singletons	Clinical—children with reading difficulties	6.1%
			
Aston Dyslexia	105 singletons	Clinical—children with reading difficulties	7.6%
			
York	103 families	Longitudinal—children with language difficulties, family history of dyslexia and typically developing	7.4% parents (*N*=174) 7.7% all probands (*N*=103) 5.5% RD group (*N*=18) 10.7% TD group (*N*=72)
			
Combined	203 singletons	Individuals scoring READ<-1.5s.d. in the above cohorts	9.6%
			
Hong Kong	220 singletons/twins	Typically developing	37.6%
			
1000 Genomes	503 singletons 504 singletons	General population	8.05% Europe 33.5% East Asia

Abbreviations: RD, reading difficulties; TD, typically developing.

a The number of probands is lower than the number of families because of missing data.

**Table 3:** Mean score of READ and VMOT stratified by the *DCDC2* deletion genotype in the Oxford dyslexia cohort

*Number of deletion alleles*	*READ (s.d.;N)*		*VMOT % (s.d.;N)*	
	*All*	*Probands*	*All*	*Probands*
0	46.2 (9.7; 417)	38.6 (6.8; 126)	18.8 (12.0; 374)	20.7 (13.5; 112)
1	45.0 (10.8; 73)	36.6 (7.6; 24)	17.5 (9.8; 66)	21.3 (14.2; 22)
2	46.4 (14.5; 7)	36.7 (8.7; 3)	16.7 (6.9; 6)	19.2 (5.8; 3)

Abbreviations: READ, single-word reading; VMOT, visual motion. The READ scores are standardized with a population mean of 50 and s.d.=10. VMOT scores are in % coherent motion at detection threshold. Poorer reading is represented by lower standard scores; reduced sensitivity to VMOT is indicated by higher scores. See Supplementary Figure 1 for the distribution of the raw scores.
