# Supplementary material for: The DCDC2 deletion is not a risk factor for dyslexia
Source: Transl Psychiatry. 2017 Jul 25;7(7):e1182–. doi: 10.1038/tp.2017.151 (PMC5538127; doi:10.1038/tp.2017.151)
Supplement: Supplementary Information [file tp2017151x1.docx]

**Supplementary Material**

**Methods**

**Visual motion (VMOT) test**

Data for the visual motion measure was available from the Oxford Dyslexia cohort and has been described previously^1^. The visual stimuli were random-dot kinematograms comprised of two fields of 300 high-luminance (80.6 cd/m^2^) white dots (1 pixel), presented on the dark background of a LCD computer display. At a fixed viewing distance of 57 cm, each patch subtended 10×14° visual angle, separated horizontally by 5°. The percentage of coherently moving dots (angular velocity=7.0 deg/s) within a given software animation frame (50 ms) was varied to the detection threshold using an adaptive psychophysical procedure^2^. The detection threshold was defined as the percentage of coherently moving dots – relative to the total number of dots present in the software animation frame – required for the detection of directional motion. The coherent dots changed direction every 1000 ms throughout the 2500 ms stimulus duration. The non-coherent dots moved in Brownian, random motion. To eliminate the possibility of detecting the direction of coherent motion by following the trajectory of a single dot, each dot had a fixed lifetime of five animation frames (250 ms).

Participants’ responses to which patch contained coherent motion was reported on a trial by trial basis by button-press. Threshold estimates were determined by calculating the geometric mean of the last six of eight reversal points within a given series of trials. Each series was repeated three times, with the mean of these individual estimates defined as the threshold. Supplementary Figure 1 shows the raw data (READ and VMOT) for each proband, stratified by *DCDC2* genotype.

The same subgroup selected for severity (N=126 families), described before both for analysis of the chromosome 6 locus^3^ and specifically for the DCDC2 deletion^4^, was analysed in this study for association with VMOT sensitivity. The definition of the subgroup derived from the observation that the chromosome 6 locus influenced the most severely affected individuals with reading disability^3,5,6^. Therefore individuals were selected when scoring ≥0.5SD below a mean measure calculated from the combined score of phonological decoding ability and orthographic coding; the two measures with the highest contributions to the linkage signal on chromosome 6 ^3^ . This selection yielded 126 families, including 313 siblings.

**Power calculations**

Power calculations were conducted using the Genetic Power Calculator ^7^ to estimate the smallest effect size that our samples could detect with 80% statistical power. The analysis was modelled for an allele frequency of 8% and 37% for the UK and Hong Kong populations, respectively. Singleton cohorts were assumed, with the exception of the Oxford Dyslexia cohort for which a sib-pairs structure was used. Alpha was set at 0.05 because of the testing of a single genetic marker. Under these assumptions the minimal effect sizes predicted to be detectable with more than 80% power was 1.5% in the combined sample (N =526) which was our largest sample. The effect sizes predicted to be detectable in the individual cohorts under the same assumptions were 2.2% in the Oxford family cohort and 8.5% in both the Aston and York cohorts and 4.3% in the Hong Kong cohort.

**Supplementary Table 1. Quantitative association analysis results for the *DCDC2* deletion in individual cohorts**

| **Cohort** | **Analysis** | **N**  **(informative)** | **Phenotype** | | **Effect size/**  **Trend*** | | **p-value** | |
| --- | --- | --- | --- | --- | --- | --- | --- | --- |
| Oxford  Families | 264 families (QTDT) | 445 | | VMOT | + | 0.36 | |  |
|  |  | 496 | | READ | - | 0.51^a^ | |  |
|  |  | 471 | | SPELL | - | 0.43 ^a^ | |  |
|  |  | 489 | | IWR | - | 0.17 ^a^ | |  |
|  |  | 492 | | PD | - | 0.08 ^a^ | |  |
|  |  | 466 | | PA | - | 0.09 ^a^ | |  |
|  | 126 families (QTDT) | 219 | | VMOT | + | 0.61 | |  |
|  |  | 253 | | READ | - | 0.05 ^a^ | |  |
|  |  | 241 | | SPELL | - | 0.09 ^a^ | |  |
|  |  | 251 | | IWR | - | 0.04 ^a^ | |  |
|  |  | 253 | | PD | - | 0.07 ^a^ | |  |
|  |  | 238 | | PA | - | 0.10 ^a^ | |  |
| Oxford  Cases |  | 272 | | READ | -1.39 | 0.40 | |  |
|  | Singletons (PLINK) | 272 | | SPELL | -0.45 | 0.80 | |  |
|  |  | 272 | | IWR | -0.45 | 0.63 | |  |
|  |  | 272 | | PD | 0.36 | 0.75 | |  |
| Aston | Singletons (PLINK) | 101 | | READ | -1.67 | 0.64 | |  |
|  |  | 101 | | SPELL | 0.17 | 0.95 | |  |
|  |  | 79 | | TOWRE | -1.17 | 0.77 | |  |
|  |  | 84 | | TOWRE_PD | -1.51 | 0.65 | |  |
|  |  | 89 | | TOWRE_SWE | -7.01 | 0.08 | |  |
| York | Singletons (PLINK) | 103 | | READ | 0.12 | 0.66 | |  |
|  |  | 102 | | SPELL | 0.19 | 0.47 | |  |
|  |  | 100 | | IWR | 0.21 | 0.38 | |  |
|  |  | 100 | | PA | 0.27 | 0.30 | |  |
|  |  | 99 | | RAN | 0.14 | 0.58 | |  |
|  |  | 102 | | TOWRE_READ | 0.08 | 0.76 | |  |
|  |  | 102 | | TOWRE_PD | 0.04 | 0.88 | |  |
|  |  | 104 | | LIT_FS | 0.13 | 0.63 | |  |
|  |  | 104 | | PHON_FS | 0.20 | 0.45 | |  |
|  |  |  | |  |  |  | |  |
|  | 103 families (QTDT) | 108 | | READ | + | 0.73 | |  |
|  |  | 108 | | SPELL | + | 0.51 | |  |
|  |  | 106 | | IWR | + | 0.38 | |  |
|  |  | 106 | | PA | + | 0.29 | |  |
|  |  | 105 | | RAN | + | 0.60 | |  |
|  |  | 107 | | TOWRE_READ | + | 0.78 | |  |
|  |  | 107 | | TOWRE_PD | + | 0.90 | |  |
|  |  | 110 | | LIT_FS | + | 0.68 | |  |
|  |  | 109 | | PHON_FS | + | 0.46 | |  |
| Hong Kong | Singletons (PLINK) | 218 | | CWR | 0.02 | 0.86 | |  |
|  |  | 161 | | COM | -0.11 | 0.41 | |  |
|  |  | 161 | | CDRAN | -0.03 | 0.77 | |  |

*Positive or negative trend indicate that the deletion is a protective or risk factor, respectively. Beta values are reported only for the analyses conducted with PLINK. ^a^Data previously published ^4^. VMOT = visual motion; READ = single word reading; SPELL = single word spelling; IWR = irregular word reading; PD = phonological decoding measured with non-word-reading; PA = phonological awareness; TOWRE = Test of Word Reading Efficiency; SWE = sight word efficiency; RAN = rapid automatic naming; LIT_FS = literacy factor score; PHON_FS = phonology factor score; CWR = Chinese word reading; COM = Chinese one minute word reading; CDRAN = Chinese Digit Rapid Automatic Naming

**Supplementary Table 2. Phenotype means in the Hong Kong cohort**

| **Phenotypes*** | **Mean (SD)** | | |
| --- | --- | --- | --- |
|  | Twins | Singletons | Total |
|  | (N=114) | (N=104) | (N=218) |
| Chinese Word Reading (CWR) | 10.4 (3.1) | 11.6 (3.1) | 11 (3.2) |
| One Minute Reading (COM) | 10.2 (3.4) | 10.4 (3.6) | 10.3 (3.5) |
| Chinese Digit Rapid Naming (CDRAN) | 9.9 (3.3) | 10.6 (3) | 10.2 (3.2) |

* The distributions for these standardised measures have a mean=10 and SD=3 ^8^

**Supplementary Table 3. Frequency of the *DCDC2* deletion (esv3608367) observed in the 1000 Genomes Project populations** ^9^

| **Population description** | **Abbreviation** | **N** | **Frequency** | **Larger population** | **Frequency (N)** |
| --- | --- | --- | --- | --- | --- |
| African Caribbeans (Barbados) | ACB | 96 | 0.01 | African (AFR) | 0.0053 (661) |
| Americans of African Ancestry (SW USA) | ASW | 61 | 0.03 |  |  |
| Esan (Nigeria) | ESN | 99 | 0 |  |  |
| Gambian (Western Divisions) | GWD | 113 | 0 |  |  |
| Luhya (Webuye, Kenya) | LWK | 99 | 0.005 |  |  |
| Mende (Sierra Leone) | MSL | 85 | 0 |  |  |
| Yoruba (Ibadan, Nigeria) | YRI | 108 | 0 |  |  |
| Colombians (Medellin, Colombia) | CLM | 94 | 0.075 | American (AMR) | 0.075 (347) |
| Mexican Ancestry (Los Angeles USA) | MXL | 64 | 0.102 |  |  |
| Peruvians (Lima, Peru) | PEL | 85 | 0.123 |  |  |
| Puerto Ricans (Puerto Rico) | PUR | 104 | 0.019 |  |  |
| Chinese Dai (Xishuangbanna, China) | CDX | 93 | 0.414 | East Asian (EAS) | 0.335 (504) |
| Han Chinese (Bejing, China) | CHB | 103 | 0.282 |  |  |
| Southern Han Chinese | CHS | 105 | 0.338 |  |  |
| Japanese (Tokyo, Japan) | JPT | 104 | 0.26 |  |  |
| Kinh (Ho Chi Minh City, Vietnam) | KHV | 99 | 0.394 |  |  |
| Utah (USA) Residents (CEPH) with Northern and Western Ancestry | CEU | 99 | 0.106 | European (EUR) | 0.0805 (503) |
| Finnish (Finland) | FIN | 99 | 0.081 |  |  |
| British (England and Scotland) | GBR | 91 | 0.066 |  |  |
| Iberian Population (Spain) | IBS | 107 | 0.061 |  |  |
| Toscani (Italy) | TSI | 107 | 0.089 |  |  |
| Bengali (Bangladesh) | BEB | 86 | 0.174 | South Asian (SAS) | 0.163 (489) |
| Gujarati Indian (Houston, Texas) | GIH | 103 | 0.136 |  |  |
| Indian Telugu (UK) | ITU | 102 | 0.191 |  |  |
| Punjabi ( Lahore, Pakistan) | PJL | 96 | 0.162 |  |  |
| Sri Lankan Tamil (UK) | STU | 102 | 0.152 |  |  |

**Supplementary Table 4. Quantitative association analysis results for the *DCDC2* deletion in combined UK samples**

| **Cohort** | **N** | **Phenotype** | **Effect size*** | **p-value** |  |
| --- | --- | --- | --- | --- | --- |
| Oxford singletons^a^ | 425 | READ | -0.10 | 0.22 | |
|  | 417 | SPELL | -0.12 | 0.20 | |
|  | 420 | IWR | -1.05 | 0.10 | |
|  | 422 | PD | -0.71 | 0.37 | |
| Oxford severe^a^ | 161 | READ | 0.01 | 0.83 | |
|  | 157 | SPELL | -0.12 | 0.30 | |
|  | 159 | IWR | -0.28 | 0.69 | |
|  | 160 | PD | 0.03 | 0.98 | |
| Oxford and Aston singletons^b^ | 526 | READ | -0.10 | 0.25 | |
|  | 518 | SPELL | -0.09 | 0.32 | |
| Oxford and Aston severe^b^ | 185 | READ | 0.03 | 0.63 | |
|  | 181 | SPELL | -0.08 | 0.49 | |

^a^Includes the Oxford cases and probands from the Oxford family cohort. ^b^Includes the Aston cases in addition to the Oxford cases and probands from the Oxford family cohort. The severe subgroups only include individuals scoring < -1.5 SD from a population mean on a single word reading measure. *Positive or negative beta indicate that the deletion is a protective or risk factor, respectively. READ = single word reading; SPELL = single word spelling; IWR = irregular word reading; PD = phonological decoding measured with non-word-reading.

**Supplementary Figures**

**
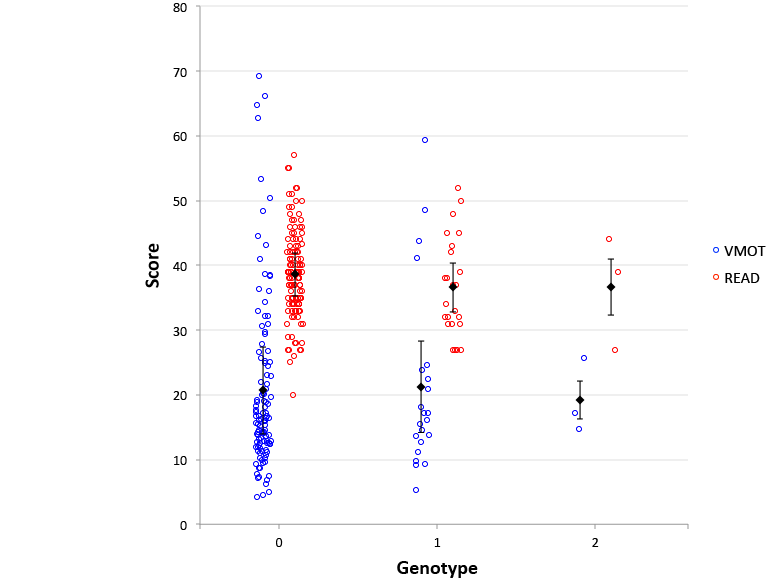
**

**Supplementary Figure 1. Individual VMOT and READ scores by genotype in the Oxford Dyslexia cohort.** Raw VMOT and READ scores for each individual shown in blue and red respectively, were stratified by the *DCDC2* deletion genotype status (0 = homozygous wildtype, 1 = heterozygous, 2 = homozygous for the *DCDC2* deletion). Mean and SD are shown in black for each variable, stratified by genotype.

**References**

1 Talcott JB, Witton C, Hebb GS, Stoodley CJ, Westwood EA, France SJ *et al.* On the relationship between dynamic visual and auditory processing and literacy skills; results from a large primary-school study. *Dyslexia* 2002; **8**: 204–225.

2 Kaernbach C. Simple adaptive testing with the weighted up-down method. *Percept Psychophys* 1991; **49**: 227–229.

3 Francks C, Paracchini S, Smith SD, Richardson AJ, Scerri TS, Cardon LR *et al.* A 77-kilobase region of chromosome 6p22.2 is associated with dyslexia in families from the United Kingdom and from the United States. *Am J Hum Genet* 2004; **75**: 1046–1058.

4 Harold D, Paracchini S, Scerri T, Dennis M, Cope N, Hill G *et al.* Further evidence that the KIAA0319 gene confers susceptibility to developmental dyslexia. *Mol Psychiatry* 2006; **11**: 1061,1085-1091.

5 Deffenbacher KE, Kenyon JB, Hoover DM, Olson RK, Pennington BF, DeFries JC *et al.* Refinement of the 6p21.3 quantitative trait locus influencing dyslexia: linkage and association analyses. *Hum Genet* 2004; **115**: 128–138.

6 Schumacher J, Anthoni H, Dahdouh F, Konig IR, Hillmer AM, Kluck N *et al.* Strong genetic evidence of DCDC2 as a susceptibility gene for dyslexia. *Am J Hum Genet* 2006; **78**: 52–62.

7 Purcell S, Cherny SS, Sham PC. Genetic Power Calculator: design of linkage and association genetic mapping studies of complex traits. *Bioinformatics* 2003; **19**: 149–150.

8 Ho CS-H, Chan D, Chung K, Tsang S-M, Lee S-H, Cheng RW-Y. *The Hong Kong Test of Specific Learning Difficulties in Reading and Writing for Primary School Students*. Second. Hong Kong: Hong Kong Specific Learning Difficulties Research Team., 2007.

9 Auton A, Abecasis GR, Altshuler DM, Durbin RM, Abecasis GR, Bentley DR *et al.* A global reference for human genetic variation. *Nature* 2015; **526**: 68–74.
